# Supplementary material for: Willingness and SARS-CoV-2 Vaccination Coverage among Healthcare Workers in China: A Nationwide Study
Source: Vaccines (Basel). 2021 Sep 6;9(9):993. doi: 10.3390/vaccines9090993 (PMC8472967; doi:10.3390/vaccines9090993)
Supplement: Supplementary file 1 [file vaccines-09-00993-s001.zip › vaccines-1307625-SI.pdf]

Table S1. The characteristics of HCWs according to the records nationally

Characteristics	Total %	Doctor %	Nurse %	Public health %	Technician %	Administrator %
**Gender**						
Male	28.2	53.8	2.3	42.1	40.4	46.1
Female	71.8	46.2	97.7	57.9	59.6	53.9
**Age group, years**						
below 30	26.9	11.3	37.5	33.7	24.1	13.6
30 to 39	32.2	28.3	34.6	35.7	33.1	25.7
40 to 49	22.4	30.3	17.5	17.2	22.5	28.3
50 or above	18.5	30.4	10.4	9.4	20.3	32.4
**Education**						
Below bachelor	24.6	17.9	30.2	26.3	21.7	21.0
Bachelor	69.9	70.1	69.7	68.0	74.9	73.9
Master or above	5.6	12.0	0.2	5.7	3.4	5.1
**Residence**						
Urban	50.9	52.9	59.0	56.3	53.6	62.0
Rural	49.1	47.1	41.0	43.7	46.4	38.0

Table S2. Vaccination Confidence, willingness and Coverage of the COVID-19 vaccine

Characteristics	Confidence rate (n, %)						Willingness (n, %)	P	Vaccination (n, %)	P
	Vaccine	P	Professionals	P	Government	p				
**Gender**										
Male	698(79.0)	0.001	764(86.4)	0.190	762(86.2)	0.435	764(86.4)	<0.001	625(70.7)	<0.001
Female	1094(72.8)		1268(84.4)		1276(85.0)		1165(77.6)		893(59.5)	
**Age group, years**										
below 30	401(64.6)	<0.001	492(79.2)	<0.001	493(79.4)	<0.001	486(78.3)	0.002	335(53.9)	<0.001
30 to 39	587(74.3)		655(82.9)		668(84.6)		616(78.0)		501(63.4)	
40 to 49	513(81.8)		575(91.7)		564(90.0)		538(85.8)		454(72.4)	
50 or above	291(83.6)		310(89.1)		313(89.9)		289(83.0)		228(65.5)	
**Education**										
High school or below	126(78.8)	0.019	141(88.1)	0.004	144(90.0)	0.001	137(85.6)	0.004	100(62.5)	0.280
Bachelor's degree	1283(76.2)		1453(86.3)		1456(86.5)		1382(82.1)		1088(64.6)	
Master's or above	383(70.7)		438(80.8)		438(80.8)		410(75.6)		330(60.9)	
**Residence**										
Urban	1364(74.9)	0.781	1542(84.7)	0.310	1545(84.9)	0.220	1457(80.1)	0.034	1185(65.1)	0.008
Rural	428(75.6)		490(86.6)		493(87.1)		472(83.4)		333(58.8)	
**Occupation**										
Doctors	430(71.3)	<0.001	496(82.3)	<0.001	497(82.4)	0.016	479(79.4)	<0.001	339(56.2)	<0.001
Nurse	237(67.7)		284(81.1)		292(83.4)		259(74.0)		201(57.4)	
Public health	770(83.3)		826(89.4)		809(87.6)		814(88.1)		706(76.4)	
Medical technician	165(70.5)		201(85.9)		209(89.3)		185(79.1)		128(54.7)	
Administrative management	190(69.1)		225(81.8)		231(84.0)		192(69.8)		144(52.4)	
**Power of post hoc**		1.00		1.00		0.60		1.00		1.00
**Total**	1792(75.1)		2032(85.2)		2038(85.4)		1929(80.8)		1518(63.6)	
**Power of post hoc**		1.00		1.00		1.00		1.00		1.00

Table S3. Reclassification and analysis of study participants through their vaccination willingness and uptake among participants without medical conditions or contraindications

Characteristics	Subgroup	Unwill & Unvac[a] (n, %)	Unwill & Vac[b] (n, %)	Willing & Unvac[c] (n, %)	Willing & Vac[d] (n, %)	P	OR(95%CI)
Gender	Female	131(11.7)	84(7.5)	91(8.2)	809(72.6)	<0.001	Ref
	Male	54(7.5)	37(5.2)	39(5.4)	588(81.9)		1.64(1.08-2.49)
Age group, years	below 30	64(13.8)	32(6.9)	66(14.2)	303(65.2)	<0.001	Ref
	30 to 39	62(10.4)	48(8)	34(5.7)	453(75.9)		3.14(2.14-4.62) ^
	40 to 49	33(6.7)	30(6)	9(1.8)	424(85.5)		NA
	40 or above	26(9.5)	11(4)	21(7.6)	217(78.9)		NA
Education	High school or below	11(9.3)	4(3.4)	7(5.9)	96(81.4)	0.007	Ref
	Bachelor's degree	112(8.6)	85(6.6)	97(7.5)	1003(77.3)		0.97(0.40-2.18)
	Master's degree or above	62(14.8)	32(7.7)	26(6.2)	298(71.3)		1.10(0.42-2.83)
Residence	Urban	154(10.7)	92(6.4)	95(6.6)	1093(76.2)	0.165	Ref
	Rural	31(7.8)	29(7.3)	35(8.8)	304(76.2)		0.91(0.58-1.44)
Occupation	Doctors	52(12.1)	26(6.1)	38(8.9)	313(73)	<0.001	Ref
	Nurse	31(12.2)	26(10.2)	23(9)	175(68.6)		1.43(0.78-2.61)
	Public health	50(6.3)	33(4.2)	33(4.2)	673(85.3)		2.43(1.46-4.04)
	Medical technician	17(10.5)	12(7.4)	17(10.5)	116(71.6)		1.04(0.55-1.97)
	Administrative management	35(17.7)	24(12.1)	19(9.6)	120(60.6)		0.80(0.43-1.49)
Vaccine confidence	No confidence in any of 3 dimensions	71(37)	43(22.4)	16(8.3)	62(32.3)	<0.001	Ref
	Confidence in any 1 of the 3 dimensions	21(26.9)	19(24.4)	5(6.4)	33(42.3)		1.75(0.56-5.48)
	Confidence in any 2 of the 3 dimensions	51(23.1)	26(11.8)	15(6.8)	129(58.4)		2.19(0.98-4.90)
	Confidence in all of the 3 dimensions	42(3.1)	33(2.5)	94(7)	1173(87.4)		2.31(1.24-4.33)
Total		185(10.1)	121(6.6)	130(7.1)	1397(76.2)		

Note: [a]: the subgroup of unvaccinated participants reported unwillingness to vaccination; [b]: the subgroup of vaccinated participants reported unwillingness to vaccination; [c]: the subgroup of unvaccinated participants reported willingness to vaccination; [d]: the subgroup of vaccinated participants reported willingness to vaccination; ^ : those aged over 30 years were combined into one group as "aged over 30 years" and introduced into the logistic regression model.

Table S4. Confidence in COVID-19 vaccine among different demographic characteristics

Characteristics	Confidence in vaccine					
	Importance	P	Safety	P	Effectiveness	P
**Gender**						
Male	734(83)	0.092	677(76.6)	<0.001	687(77.7)	<0.001
Female	1204(80.2)		1039(69.2)		1065(70.9)	
**Age group, years**						
below 30	482(77.6)	0.008	367(59.1)	<0.001	393(63.3)	<0.001
30 to 39	634(80.3)		562(71.1)		568(71.9)	
40 to 49	528(84.2)		506(80.7)		506(80.7)	
50 or above	294(84.5)		281(80.7)		285(81.9)	
**Education**						
High school or below	140(87.5)	0.003	117(73.1)	0.433	123(76.9)	0.007
Bachelor's degree	1381(82)		1221(72.5)		1259(74.8)	
Master's or above	417(76.9)		378(69.7)		370(68.3)	
**Residence**						
Urban	1478(81.2)	0.973	1316(72.3)	0.454	1327(72.9)	0.327
Rural	460(81.3)		400(70.7)		425(75.1)	
**Occupation**						
Doctors	464(76.9)	<0.001	417(69.2)	<0.001	414(68.7)	<0.001
Nurse	271(77.4)		220(62.9)		232(66.3)	
Public health	797(86.3)		743(80.4)		749(81.1)	
Medical technician	188(80.3)		158(67.5)		162(69.2)	
Administrative management	218(79.3)		178(64.7)		195(70.9)	
**Total**	1938(81.2)		1716(71.9)		1752(73.4)	

Table S5. Confidence in professional institutes and government among different demographic characteristics

Characteristics	Professionals						Government	P
	Healthcare	P	Hospital/CDC	P	Manufacture	P		
**Gender**								
Male	754(85.3)	0.166	775(87.7)	0.174	732(82.8)	0.094	762(86.2)	0.435
Female	1248(83.1)		1286(85.6)		1201(80)		1276(85)	
**Age group, years**								
below 30	483(77.8)	<0.001	498(80.2)	<0.001	461(74.2)	<0.001	493(79.4)	<0.001
30 to 39	645(81.6)		671(84.9)		629(79.6)		668(84.6)	
40 to 49	566(90.3)		579(92.3)		547(87.2)		564(90)	
50 or above	308(88.5)		313(89.9)		296(85.1)		313(89.9)	
**Education**								
High school or below	139(86.9)	0.018	143(89.4)	0.012	131(81.9)	0.011	144(90)	0.001
Bachelor's degree	1429(84.9)		1470(87.3)		1387(82.4)		1456(86.5)	
Master's or above	434(80.1)		448(82.7)		415(76.6)		438(80.8)	
**Residence**								
Urban	1525(83.8)	0.844	1569(86.2)	0.726	1464(80.4)	0.220	1545(84.9)	0.220
Rural	477(84.3)		492(86.9)		469(82.9)		493(87.1)	
**Occupation**								
Doctors	492(81.6)	<0.001	501(83.1)	0.001	477(79.1)	0.001	497(82.4)	0.016
Nurse	281(80.3)		292(83.4)		268(76.6)		292(83.4)	
Public health	814(88.1)		832(90)		787(85.2)		809(87.6)	
Medical technician	195(83.3)		204(87.2)		188(80.3)		209(89.3)	
Administrative management	220(80)		232(84.4)		213(77.5)		231(84)	
**Total**	2002(83.9)		2061(86.4)		1933(81.0)		2038(85.4)	

Table S6. Willingness rate of the COVID-19 vaccination grouped by vaccine confidence

Characteristics	Total (n=2386)	Doctor (n=603)	Nurse (n=350)	CDC (n=924)	Tech (n=234)	Admin (n=275)	P-value
**Total**	1929(80.8)	479(79.4)	259(74.0)	814(88.1)	185(79.1)	192(69.8)	<0.001
**Trust in the importance of vaccination**							
Disagree	218(48.7)	65(46.8)	38(48.1)	72(56.7)	21(45.7)	22(38.6)	0.202
Agree	1711(88.3)	414(89.2)	221(81.5)	742(93.1)	164(87.2)	170(78.0)	<0.001
**Confidence on vaccine safety**							
Disagree	340(50.7)	88(47.3)	59(45.4)	113(62.4)	43(56.6)	37(38.1)	0.001
Agree	1589(92.6)	391(93.8)	200(90.9)	701(94.3)	142(89.9)	155(87.1)	0.005
**Confidence on vaccine effectiveness**							
Disagree	335(52.8)	98(51.9)	59(50)	100(57.1)	45(62.5)	33(41.3)	0.067
Agree	1594(91.0)	381(92)	200(86.2)	714(95.3)	140(86.4)	159(81.5)	<0.001
**Confidence on healthcare provider**							
Disagree	179(46.6)	48(43.2)	27(39.1)	63(57.3)	22(56.4)	19(34.5)	0.019
Agree	1750(87.4)	431(87.6)	232(82.6)	751(92.3)	163(83.6)	173(78.6)	<0.001
**Confidence on CDCs & hospitals**							
Disagree	143(44.0)	42(41.2)	22(37.9)	51(55.4)	14(46.7)	14(32.6)	0.076
Agree	1786(86.7)	437(87.2)	237(81.2)	763(91.7)	171(83.8)	178(76.7)	<0.001
**Confidence on vaccine manufacturers**							
Disagree	211(46.6)	56(44.4)	35(42.7)	77(56.2)	22(47.8)	21(33.9)	0.042
Agree	1718(88.9)	423(88.7)	224(83.6)	737(93.6)	163(86.7)	171(80.3)	<0.001
**Confidence on the government**							
Disagree	145(41.7)	44(41.5)	20(34.5)	60(52.2)	10(40.0)	11(25.0)	0.021
Agree	1784(87.5)	435(87.5)	239(81.8)	754(93.2)	175(83.7)	181(78.4)	<0.001

Note: CDC, employees belong to public health; Tech, employees belong to medical technician; Admin, employees belong to administrative management.

Table S7. Supplemental Table Logistic regression on willingness among **Doctors**

	**Willingness among Doctors**		
**Characteristics**	**OR**	**95%CI**	**P**
**Gender**			
Male	Ref.		0.035
Female	0.56	0.33-0.96	
**Age group, years**			
below 30	Ref.		0.977
30 to 39	0.91	0.48-1.74	0.777
40 to 49	0.93	0.45-1.92	0.841
50 or above	0.82	0.33-2.03	0.665
**Education**			
High school or below	Ref.		0.263
Bachelor's degree	0.17	0.02-1.50	0.109
Master's or above	0.18	0.02-1.80	0.145
**Residence**			
Urban	Ref.		0.195
Rural	1.58	0.79-3.15	
**Confidence**			
Importance of vaccination	2.69	1.50-4.83	0.001
Vaccine safety	6.45	3.39-12.26	<0.001
Vaccine effectiveness	1.57	0.80-3.07	0.186
Confidence in professionals	1.10	0.50-2.42	0.814
Confidence in government	2.42	1.15-5.08	0.020

Table S8. Supplemental Table Logistic regression on willingness among **Nurses**

**Characteristics**	**Willingness among Nurse**		
	**OR**	**95%CI**	**P**
**Gender**			
Male	Ref.		0.059
Female	0.29	0.08-1.05	
**Age group, years**			
below 30	Ref.		0185
30 to 39	0.44	0.21-0.93	0.031
40 to 49	0.50	0.19-1.28	0.147
50 or above	0.59	0.16-2.19	0.431
**Residence**			
Urban	Ref.		0.390
Rural	1.32	0.70-2.50	
**Confidence**			
Importance of vaccination	0.74	0.32-1.70	0.471
Vaccine safety	9.52	4.17-21.76	<0.001
Vaccine effectiveness	1.34	0.58-3.05	0.493
Confidence in professionals	1.19	0.42-3.34	0.740
Confidence in government	2.87	1.03-8.04	0.045

Table S9. Supplemental Table Logistic regression on willingness among **Public health employees**

	Willingness among Public health employees		
**Characteristics**	**OR**	**95%CI**	**P**
**Gender**			
Male	Ref.		0.026
Female	0.56	0.34-0.93	
**Age group, years**			
below 30	Ref.		0.060
30 to 39	0.80	0.41-1.57	0.516
40 to 49	0.97	0.48-1.99	0.942
50 or above	0.39	0.18-0.85	0.018
**Education**			
High school or below	Ref.		0.205
Bachelor's degree	0.96	0.34-2.74	0.938
Master's or above	0.58	0.18-1.84	0.352
**Residence**			
Urban	Ref.		0.972
Rural	0.99	0.51-1.91	
**Confidence**			
Importance of vaccination	2.33	1.22-4.48	0.011
Vaccine safety	2.21	1.17-4.15	0.014
Vaccine effectiveness	5.84	3.08-11.11	<0.001
Confidence in professionals	0.33	0.12-0.91	0.031
Confidence in government	3.72	1.46-9.50	0.006

Table S10. Supplemental Table Logistic regression on willingness among **Medical technicians**

Characteristics	Willingness among Medical technicians		
	**OR**	**95%CI**	**P**
**Gender**			
Male	Ref.		
Female	0.95	0.43-2.13	0.905
**Age group, years**			
below 30	Ref.		0.316
30 to 39	0.45	0.17-1.14	0.092
40 to 49	0.43	0.15-1.30	0.136
50 or above	0.42	0.10-1.69	0.223
**Education**			
High school or below	Ref.		0.149
Bachelor's degree	0.19	0.02-2.04	0.170
Master's or above	0.11	0.01-1.29	0.078
**Residence**			
Urban	Ref.		
Rural	0.66	0.25-1.74	0.400
**Confidence**			
Importance of vaccination	4.25	1.64-10.98	0.003
Vaccine safety	4.25	1.64-11.02	0.003
Vaccine effectiveness	0.66	0.24-1.86	0.434
Confidence in professionals	1.85	0.46-7.50	0.390
Confidence in government	1.10	0.26-4.61	0.898

Table S11. Supplemental Table Logistic regression on willingness among **Administrators**

Characteristics	Willingness among Administrators		
	**OR**	**95%CI**	**P**
**Gender**			
Male	Ref.		
Female	0.59	0.29-1.18	0.905
**Age group, years**			
below 30	Ref.		0.378
30 to 39	0.68	0.30-1.53	0.349
40 to 49	1.06	0.41-2.77	0.901
50 or above	0.44	0.14-1.35	0.150
**Education**			
High school or below	Ref.		0.024
Bachelor's degree	0.24	0.06-0.92	0.037
Master's or above	0.13	0.03-0.57	0.017
**Residence**			
Urban	Ref.		
Rural	0.81	0.33-1.96	0.639
**Confidence**			
Importance of vaccination	2.28	1.02-5.09	0.044
Vaccine safety	5.89	2.57-13.51	<0.001
Vaccine effectiveness	1.02	0.41-2.55	0.970
Confidence in professionals	0.89	0.28-2.79	0.841
Confidence in government	4.14	1.29-13.28	0.017

Table S12. Supplemental Table Logistic regression on willingness among **total sample**

**Characteristics**	**Willingness among Total sample**		
	**OR**	**95%CI**	**P**
**Gender**			
Male	Ref.		<0.001
Female	0.53	0.40-0.69	
**Age group, years**			
below 30	Ref.		0.020
30 to 39	0.67	0.49-0.92	0.012
40 to 49	0.85	0.59-1.22	0.371
50 or above	0.57	0.37-0.87	0.566
**Education**			
High school or below	Ref.		0.128
Bachelor’s degree	0.82	0.48-1.42	0.479
Master’s or above	0.62	0.34-1.13	0.119
**Residence**			
Urban	Ref.		.386
Rural	1.15	0.84-1.57	
**Confidence**			
Importance of vaccination	2.07	1.52-2.81	<0.001
Vaccine safety	4.94	3.60-6.78	<0.001
Vaccine effectiveness	1.88	1.35-2.61	<0.001
Confidence in professionals	0.92	0.60-1.42	0.701
Confidence in government	2.18	1.45-3.28	<0.001
